# Supplementary material for: Spatial organization in microbial range expansion emerges from trophic dependencies and successful lineages
Source: Commun Biol. 2020 Nov 18;3:685. doi: 10.1038/s42003-020-01409-y (PMC7674409; doi:10.1038/s42003-020-01409-y)
Supplement: Supplementary file 1 — Supplementary information [file 42003_2020_1409_MOESM1_ESM.pdf]

Supplementary information:

Spatial organization in microbial range expansion emerging from trophic dependencies and successful lineages

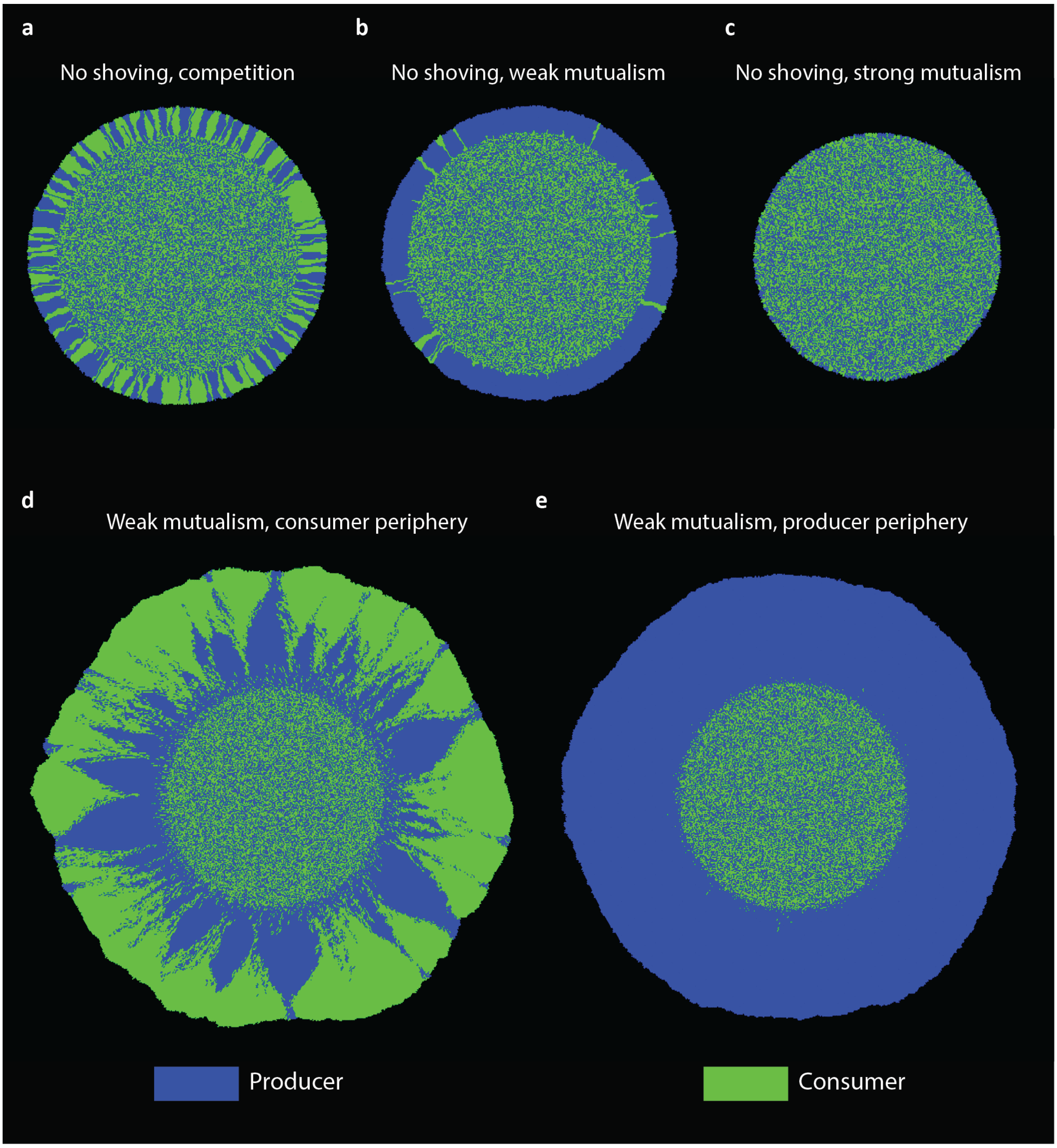

SI Figure 1. Importance of bacterial shoving and initial spatial positioning for pattern formation. Removal of the shoving algorithm (i.e. only cells at the colony periphery can contribute to colony expansion through placing their progeny on unoccupied grid nodes) results in a similar pattern (although reduce in expansion) for the competitive scenario (a), a suppression of super sectors in case of weak mutualism (b) and a significantly reduced expansion rate in the strong mutualistic scenario (c). In case of weak mutualistic conditions, initial positioning of consumer cells is fundamental for colony pattern formation. When the whole periphery is occupied by consumer cells (d), numerous consumer super branches emerge since consumer cells are frequently pushed ahead by the proliferating producer. When the inoculated colony periphery is dominated by producer cells (e), no consumer branches emerge since the consumer is buried behind the advancing producers.

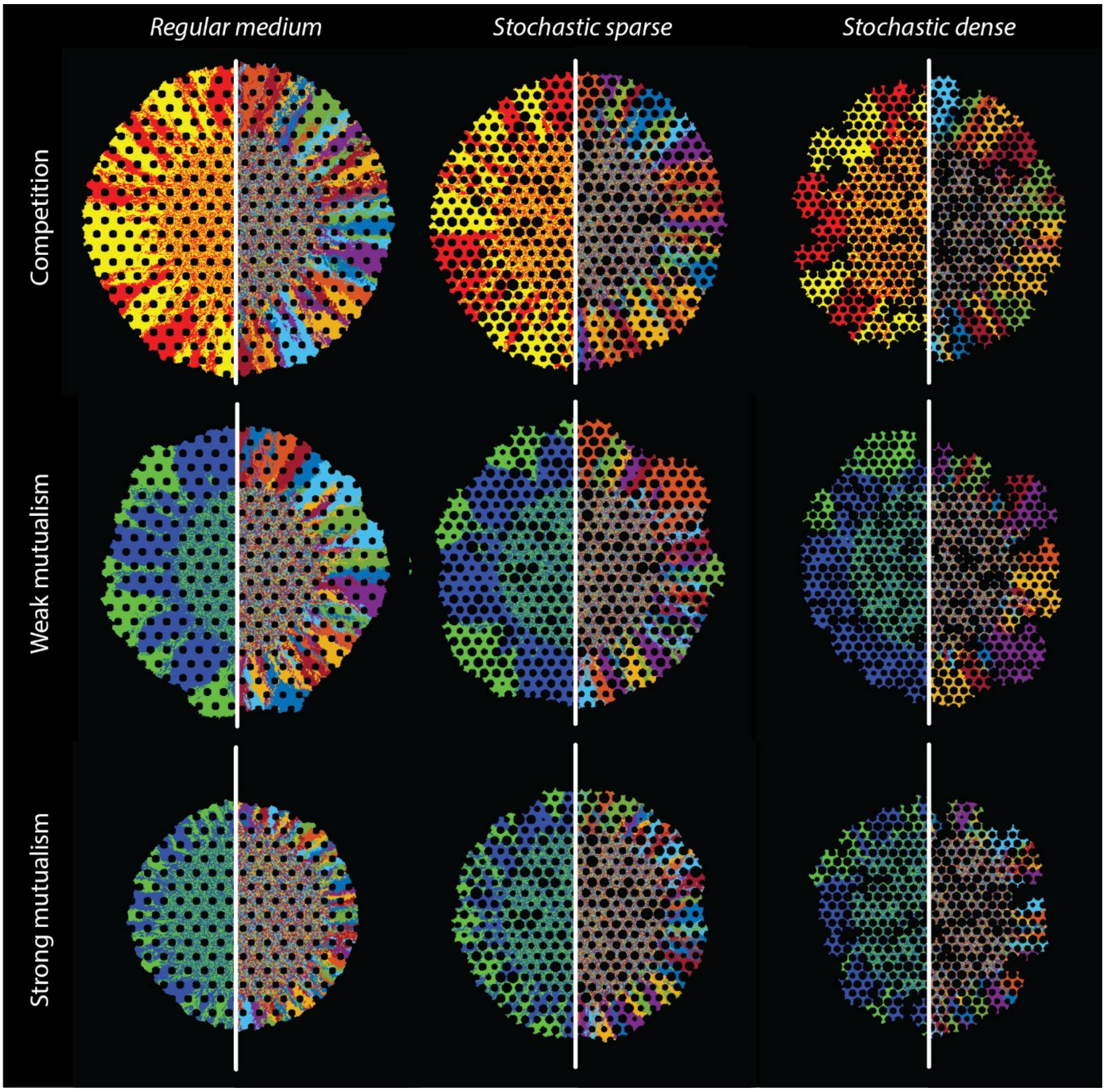

SI Figure 2. Inclusion of stochastic variations in particle size simulations of structured habitats (with different obstacle spacing). The inclusion of additional heterogeneity through stochastic variation of obstacle diameter whilst keeping the spacing equal results in an amplified effect of lineage coalescence for all scenarios and a reduction in observed consumer branches in the weak and strong mutualistic scenarios. Quantification of total peripheral lineages, peripheral consumer branches and biomass is shown in SI Table 1.

SI Table 1: Quantification of total number of peripheral lineages [#], persistent consumer branches [#] and biomass [10^{-8} kg] for competition (CT), weak mutualism (WM) and strong mutualism (SM) in all simulated habitat scenarios. Table shows mean values of all replicates including standard deviations in brackets.

	Total lineages [#]			*Consumer branches [#]*			*Biomass [kg]*		
	CT	*WM*	*SM*	*CT*	*WM*	*SM*	*CT*	*WM*	*SM*
Homogeneous	163 (13)	143 (12)	283 (12)	85 (7)	22 (6)	110 (5)	3.43 (0.05)	3.18 (0.10)	1.74 (0.01)
Regular coarse	107 (3)	89 (7)	173 (4)	52 (2)	27 (6)	68 (5)	2.96 (0.02)	2.70 (0.06)	1.56 (0.01)
Regular medium	87 (6)	73 (6)	134 (18)	44 (6)	19 (3)	51 (8)	2.56 (0.24)	2.31 (0.22)	1.37 (0.02)
Regular fine	68 (4)	65 (5)	100 (5)	33 (6)	14 (4)	36 (3)	1.91 (0.03)	1.71 (0.03)	1.03 (0.01)
Stochastic coarse	100 (5)	86 (8)	175 (8)	49 (4)	25 (8)	66 (4)	2.95 (0.03)	2.69 (0.06)	1.55 (0.01)
Stochastic medium	85 (3)	73 (6)	143 (7)	40 (5)	23 (6)	54 (4)	2.64 (0.03)	2.37 (0.03)	1.36 (0.01)
Stochastic fine	62 (4)	56 (3)	98 (3)	30 (5)	15 (2)	38 (5)	1.85 (0.05)	1.62 (0.04)	1.00 (0.02)
